# Chronic pain and hypertension and mediation role of inflammation and depression

**DOI:** 10.1161/HYPERTENSIONAHA.125.25544

**Published:** 2025-11-17

**Authors:** Pei Qin, Frederick K. Ho, Carlos A. Celis-Morales, Jill P. Pell

**Affiliations:** 1 School of Health and Wellbeing, University of Glasgow, Glasgow UK; 3 School of Cardiovascular and Metabolic Health, University of Glasgow, Glasgow UK; 4 Human Performance Lab, Education, Physical Activity and Health Research Unit, University Católica del Maule, Talca, Chile; 5 Centro de Investigación en Medicina de Altura (CEIMA), Universidad Arturo Prat, Iquique, Chile

**Keywords:** chronic pain, chronic musculoskeletal pain, pain locations, hypertension, cohort

## Abstract

**Background:**

The association between chronic pain and incident hypertension is unclear. This study aimed to investigate the associations between different pain characteristics (pain type, location and spread) and incident hypertension, and whether they were mediated by inflammation, depression or medication.

**Methods:**

In a cohort study of 206,963 UK Biobank participants, multivariable Cox-proportional regression was used to investigate the associations between pain at baseline, measured via a touchscreen questionnaire, and incident hypertension ascertained from linkage to health records. Mediation analysis was conducted to estimate the percentage of the association mediated by inflammation, depression and medications.

**Results:**

Over a median follow-up of 13.5 years, 19,911 (9.62%) participants developed hypertension. Compared with no pain, those with short-term pain (hazard ratio [HR] 1.10, 95% confidence interval [CI], 1.03–1.17), chronic localized pain (HR 1.20, 95% CI 1.14–1.26) and chronic widespread pain (HR 1.75, 95% CI 1.52–2.00) had increased risk of hypertension. A dose-relationship also existed between the number of chronic pain sites and hypertension. Chronic headache, neck, back, abdominal, hip and widespread pain were all associated with increased risk of hypertension, as was chronic musculoskeletal pain. Together, depression (11.3%) and inflammation (0.4%), as measured by C-reactive protein, mediated 11.7% of the association between chronic pain and hypertension.

**Conclusions:**

People with chronic pain are at higher risk of developing hypertension, and this is partly explained by inflammation and depression. These findings reinforce the need for pain management, and the monitoring and early detection of hypertension.

## Graphical abstract

**Figure:**
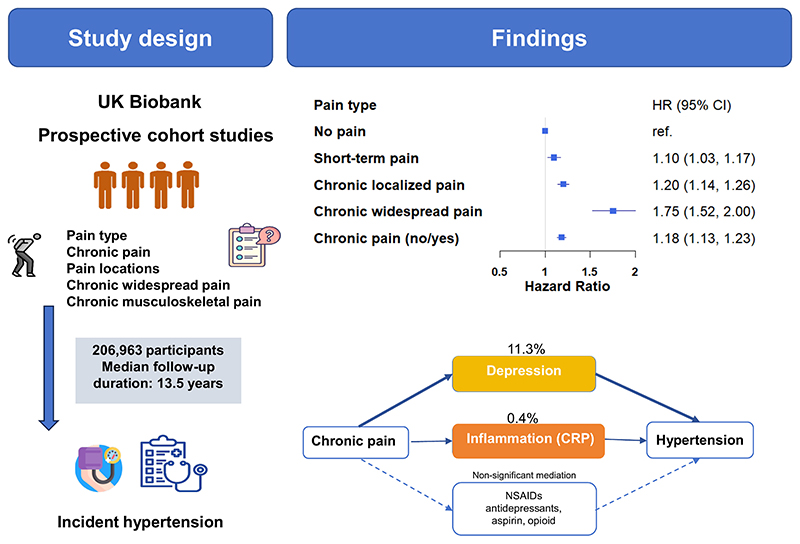

## Abbreviations

BMI: body mass index
CVD: cardiovascular diseases
DBP: diastolic blood pressure
HDL: high-density lipoprotein
HbA1c: glycated haemoglobin
LDL: low-density lipoprotein
NSAID: nonsteroidal anti-inflammatory drugs
SBP: systolic blood pressure
SD: standard deviation
TG: triglyceride
WC: waist circumference

## Introduction

Hypertension is major risk factor for cardiovascular diseases (CVD) and mortality (1). The number of people living with hypertension has doubled between 1990 and 2019, reaching 1.3 billion worldwide, with a global age-standardised prevalence among adults of approximately 34% (2). Considering the large burden of hypertension, it is important to identify risk factors in order to inform prevention, diagnosis, and treatment strategies.

Chronic pain, defined as pain lasting longer than 3 months by the International Association for the Study of Pain, is one of the top five underlying causes of years lived with disability globally (3). It affects around 20–30% of the general adult population globally (3); 35%~60% in Europe (4, 5) and 35.0%~51.3% in the UK. There is a wealth of evidence on the role of chronic pain in the development of atherosclerosis (6), CVD (7, 8), and all-cause mortality (9); however, the relationship between chronic pain and blood pressure has been contradictory (10) and studies on pain and hypertension and, are very limited in number (11–13) and their findings are not consistent. Evidence is also lacking on the associations between pain type, duration and location and hypertension and no study, to our knowledge, has explored the dose-relationship between the number of chronic pain sites and incident hypertension. Meanwhile, very few studies have investigated the associations between specific pain sites and hypertension, and existing studies have focused on shoulder (12) and abdominal(13) pain.

Chronic musculoskeletal pain is the most common type of chronic pain in the general population (14), with a prevalence of approximately 35.7% (15), and has been shown to be associated with limited activity, poor quality of life, and disability (16). Increasing evidence has suggested an association between chronic musculoskeletal pain and metabolic factors (17), low physical activity (18), and cardiovascular disease (19). Nevertheless, research on the association between chronic musculoskeletal pain and hypertension is lacking.

Given that inflammation has been linked to both pain and the risk of hypertension (20) (21), inflammatory markers may serve as intermediate markers of hypertension risk linked to chronic pain. Patients with chronic pain have a higher risk of depression (22), which may further increase the risk of hypertension (23), suggesting a potential mechanism mediating the association between chronic pain and risk of hypertension. However, to date, no studies have examined the extent to which the association between pain and hypertension is mediated through inflammation and depression. The medications prescribed to manage chronic pain, such as nonsteroidal anti-inflammatory drugs (NSAIDs) and antidepressants, may themselves increase the risk of hypertension (24–26). A better understanding of the roles of inflammation, depression, and pain medication may inform preventive and treatment interventions by identifying modifiable factors on the pathway from pain to hypertension.

Therefore, large evidence gaps currently exist in our understanding of the associations between the type, location, spread of pain and hypertension and the potential mediating role of inflammation, depression, and pain medication. To address this evidence gap, this study used UK Biobank data to investigate the associations between different pain characteristics (pain type and spread, and number of sites) and incident hypertension and whether inflammation, depression and pain medications mediated these associations. In addition, we explored the associations with chronic musculoskeletal pain specifically.

## Methods

### Study sample and participants

A population cohort study was conducted using data from UK Biobank which recruited over 500,000 participants at 22 assessment centres across England, Scotland and Wales between 2006 and 2010. Data were collected at the baseline visit via a touch-screen questionnaire, interview, physical measurements, and biological samples (27). The UK Biobank study received ethical approval the North-West Multi-centre Research Ethics Committee (ref: 11/NW/0382) and all participants provided electronic informed consent in accordance with the Declaration of Helsinki.

### Pain

The pain characteristics of interest included the type and locations of pain and number of pain sites (8); overall and for musculoskeletal pain specifically. In the baseline touch-screen questionnaire, participants were asked whether they had experienced pain in the last month, that interfered with their usual activities, in the head, face, neck/shoulder, back, stomach/abdomen, hip, knee, or all over their body. If pain was reported, they were asked if it had persisted for more than three months. Based on these responses, chronic pain (yes or no) was defined as pain lasting at least three months at one or more body site or all over the body. For each body site, pain was categorized into free of pain, short-term pain (pain in the last month but less than three months duration) or chronic pain (pain of at least three months duration). The total number of sites with chronic pain was summed then classified as: 0, 1, 2-3 and ≥4 chronic pain sites. Finally, pain type was classified as: free of pain, short-term pain (pain at one or more body site within the last month, but less than three months duration), chronic localized pain (pain at one or more body site of at least three months duration) and chronic widespread pain (pain “all over the body” for at least three months duration). Chronic musculoskeletal pain was defined as having any pain of at least three months duration in the hip, knee, back, or neck/shoulder. The number of affected sites was summed and categorized as: 0, 1, 2-3, and 4 chronic musculoskeletal pain sites.

### Hypertension

The outcome, hypertension, was ascertained from hospital admission and day case records and defined using International Statistical Classification of Diseases and Related Health Problems, Tenth Revision (ICD-10) codes (I10-I13, I15). Follow-up duration was calculated from the baseline date to the date of a recorded diagnosis of hypertension, death, or censoring, whichever occurred first.

### Covariates

The covariates were selected based on previous evidence of their associations with both pain and hypertension (28, 29). Demographic and lifestyle data were self-reported at baseline using the touch-screen questionnaire and interview: age, sex (male or female), ethnicity (white, South Asian, or other), smoking status (never, former or current smoker), alcohol consumption (units/week), physical activity (low, moderate, high), sedentary behaviour (hours/day), fruit and vegetable intake (portions per day), and sleep duration (1-6, 7-8 or ≥9 hours/day). Townsend deprivation index was calculated from postcode of residence(30). The number of prevalent long-term conditions was calculated by summing 42 self-reported doctor-diagnosed conditions reported at baseline (e.g., asthma, coronary heart disease, atrial fibrillation, dyspepsia, diabetes, thyroid disorders, chronic obstructive pulmonary disease, chronic kidney disease, heart failure, chronic liver disease) (31) and categorized into: 0, 1, or ≥2 (32).

Physical and biological measurements included height and weight (used to derive body mass index (BMI)), waist circumference, systolic and diastolic blood pressure, and serum total, low-density lipoprotein (LDL), and high-density lipoprotein (HDL) cholesterol and triglycerides, and hemoglobin A1c (HbA1c). Use of cholesterol lowering and antihypertensive medication use and insulin therapy was self-reported. Additional details about these measurements can be found in the UK Biobank online protocol (https://www.ukbiobank.ac.uk/).

### Mediators

Depression was ascertained from the Recent Depressive Symptoms (RDS-4) tool administered through the touchscreen questionnaire. Participants reported the frequency of depressed mood, unenthusiasm/disinterest, tenseness/restlessness, and tiredness/lethargy in the previous two weeks as not at all, several days, more than half the days, nearly every day. The Likert scales were scored from 1 to 4 and summed over the four questions to produce an overall RDS-4 score that ranged from 4 to 16. Serum C-reactive protein (CRP) concentration (mg/L) was measured by immunoturbidimetric high-sensitivity analysis on a Beckman Coulter AU5800. Medications included opioids, statin use, antidepressants, and non-steroidal anti-inflammatory drugs (NSAID) and were self-reported at baseline using the touchscreen questionnaires and confirmed at interview.

### Statistical analyses

Baseline characteristics of the study population were summarized as mean and standard deviation (SD) for continuous variables that were approximately normally distributed, median and interquartile range (IQR) for skewed distributions, and frequency with percentage (%) for categorical variables. Group differences in baseline characteristics were assessed using analysis of variance, Mann–Whitney U, and χ^2^ tests, respectively.

Hazard ratios (HRs) and their 95% confidence intervals (CIs) were estimated from Cox proportional hazard models of the associations between pain variables and incident hypertension. The pain variables, entered into separate models, were pain type (categorical), chronic pain (binary), number of chronic pain sites (ordinal), pain location (categorical), chronic musculoskeletal pain (binary), and number of chronic musculoskeletal pain sites (ordinal). The proportional hazard assumptions were tested using Schoenfeld residuals and log–log survival plots. Four models were run for each of the pain exposures: Model 1 adjusted for age, sex, Townsend deprivation index, and ethnicity; Model 2 additionally adjusted for smoking status, alcohol consumption, physical activity, total sedentary time, sleep duration, and fruit and vegetable intake; Model 3 additionally adjusted for HDL and total cholesterol, systolic blood pressure, HbA1c, number of long term conditions, and use of cholesterol-lowering medications, and insulin therapy; Model 4 (fully adjusted) additionally adjusted for use of antidepressant medications, opioids, aspirin and other NSAIDs. The risk of hypertension per one-site increment in the number of chronic pain and chronic musculoskeletal pain sites was also estimated.

We assessed the potential mediating role of inflammatory factors (CRP), depression (RDS-4 score), and pain medications on the association between chronic pain and incident hypertension, in the presence of the mediator-outcome confounding variables. First, the mediating effect of each mediator on the association was analyzed in simple mediation. Second, indicators with statistically significant indirect effects were included in the same mediation model to calculate the combined mediation effect. The mediation analyses included the same covariates included in the main Model 3. The 95% confidence intervals of our estimates were generated using the percentile bootstrapping inference method, with 1,000 bootstraps in each procedure and a random seed for reproducibility purposes. Mediation analyses were conducted using the CMAverse R studio package (33). To further check the sensitivity of the estimates for the mediation analysis, we calculated E-values that indicate the minimum strength of an unobserved confounding factor that would invalidate the observed association (34). E value scores were categorised as small (HR <1.25), medium (HR 1.25–2.00), or large (HR >2.00). “EValues” packages in R was used to calculated E-values of total effect, direct effect and indirect effect.

The analysis were performed using the complete case analysis. All analyses were conducted using R, version 4.3.2 (R Foundation for Statistical Computing) statistical packages. Two-tailed p-values <0.05 were considered to indicate significance.

## Results

### Participant baseline characteristics

**Figure 1:**
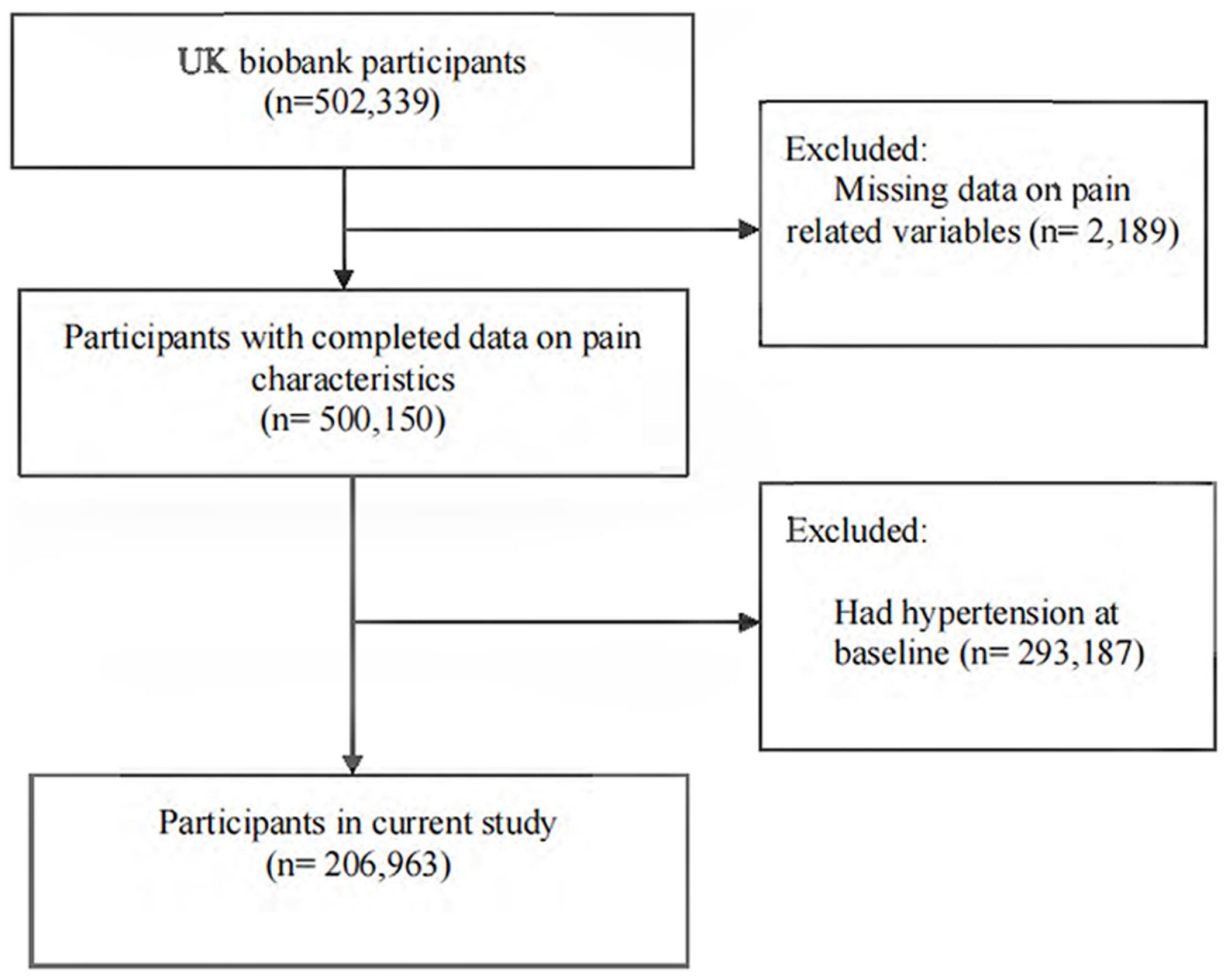
Flowchart of participant inclusion.

Overall, 295,376 participants were excluded from the study: 2,189 because they had missing data on pain-related variables at baseline, and 293,187 because they had hypertension at baseline ascertained through self-reported physician diagnosis, systolic blood pressure ≥140 mmHg, diastolic blood pressure ≥90 mmHg, or use of antihypertensive medication. Hence, 206,963 individuals were included (Figure 1).

A total of 206,963 participants were included in the study. Their mean age was 53.84 years (standard deviation [SD] 8.07), 61.7% were women, and 96.7% were of white ethnicity (Table 1). At baseline, 82,869 (40.1%) reported no pain, 36,654 (17.7%) short-term pain, 85,118 (41.1%) chronic localized pain (1-7 sites), and 2,322 (1.1%) chronic widespread pain. Among all participants, 72,912 (35.2%) experienced chronic musculoskeletal pain: 45,361 (62.2%) at 1 site, 25,439 (34.9%) at 2-3 chronic musculoskeletal pain sites, and 2,112 (3.2%) at 4 sites.

Baseline characteristics for the whole study population and stratified by chronic pain and pain type are described in Table 1. Compared to those without chronic pain, participants with chronic pain were more likely to be female, lived in more deprived areas, and had unhealthier lifestyles, larger waist circumferences and higher BMI, and more long-term conditions. They had higher concentrations of total and LDL cholesterol and triglycerides, lower HDL cholesterol and were more likely to take cholesterol-lowering medications, antidepressive medications, aspirin, opioids, and NSAIDs. A similar pattern was observed with increasing number of chronic pain sites.

### Pain type and hypertension

Over a median follow-up period of 13.5 years, 19,911 (9.6%) individuals developed hypertension. After adjustment for all covariates, compared to those without pain, participants with short-term pain (HR, 1.10; 95% CI, 1.03–1.17) and chronic localized pain (HR, 1.20; 95% CI, 1.14–1.26) had a higher risk of incident hypertension, while people with chronic widespread pain had the highest risk of incident hypertension (HR, 1.75; 95% CI, 1.52–2.00) (Table 2).

### Chronic pain and number of chronic pain sites and hypertension

In the fully adjusted models, chronic pain was associated with a higher risk of hypertension (HR 1.18; 95% CI, 1.13–1.23), compared to those without chronic pain (Table 2). The risk of hypertension increased with the number of chronic pain sites (1 site: HR 1.09, 95% CI, 1.03–1.15; 2-3 sites: HR 1.24, 95% CI, 1.17–1.31; ≥4 sites: HR 1.31, 95% CI, 1.19–1.45) (Table 3). The risk of hypertension increased by 7% (*P*_trend_<0.001) per one site increase in chronic pain.

### Chronic musculoskeletal pain and number of chronic musculoskeletal pain sites and hypertension

Chronic musculoskeletal pain was associated with higher risk of incident hypertension in the fully adjusted model (HR 1.07; 95% CI, 1.05–1.10). Risk of hypertension increased with the number of chronic musculoskeletal pain sites (Table 3) with evidence, from the trend analysis, of a dose-relationship.

### Specific pain sites and hypertension

Compared to participants who had no pain, participants reporting chronic widespread pain had the highest risk of incident hypertension, followed by chronic abdominal pain (HR 1.43; 95% CI 1.20, 1.71), chronic headache (HR 1.22; 95% CI 1.13, 1.31), then chronic neck (HR 1.19; 95% CI 1.11, 1.28), chronic hip (HR 1.17; 95% CI 1.01, 1.34) and chronic back (HR 1.16; 95% CI 1.07, 1.25) pain (Table 4); participants with. short-term headache (HR 1.15; 95% CI 1.07, 1.23), back (HR 1.24; 95% CI 1.12, 1.37), and neck/shoulder pain (HR 1.15; 95% CI 1.04, 1.28) also reported increased risk.

### Mediation analyses

The association between chronic pain and risk of hypertension was significantly mediated by CRP and depression (RDS-4 score); each mediating between 0.4% and 11.3% of the association. CRP and depression (RDS-4 score) together explained 11.7% (7.65, 17.4) of the association between chronic pain and risk of hypertension (Table 5). There was no evidence of significant indirect mediation of pain by opiods, NSAIDs, antidepressants, or aspirin.

## Sensitivity analyses

E-values were calculated to estimate the potential impact of residual confounding on our results (Table S1). The mediational E values for the average causal mediation effects on the risk ratio scale were 1.148 for depression, 1.025 for CRP, and 1.153 for CRP and depression together in the mediation models. This means that the strength of the association of an unmeasured confounder with both the mediator (depression) and hypertension, conditional on the measured covariates (identified confounders), would need to be, at minimum, greater than 1.148 on the risk ratio scale to reduce the average causal mediation effect to 0 (no effect). The strength of the association of an unmeasured confounder with both CRP and hypertension, conditional on the measured covariates, would need to be greater than 1.025 on the risk ratio scale to reduce the average causal mediation effect to 0.

## Discussion

In a large-scale population cohort, chronic pain was associated with incident hypertension, independent of demographic, lifestyle, and health confounders. Consistent dose-response relationships were found in terms of the number of body sites affected by chronic pain and chronic musculoskeletal pain, and across the spectrum from no pain, short-term pain, and chronic localized pain to chronic widespread pain. Pain in different sites was associated with different effect sizes. The risk of hypertension was highest for chronic widespread pain followed by chronic abdominal pain, headache and neck pain. Depression, as measured by RDS-4, and inflammation, as measured by CRP concentration, together explained 11.7% of the association between chronic pain and risk of hypertension; of the two significant mediators, depression accounted for a large proportion of the mediation effect. In contrast, there was no evidence that the association was mediated by medication.

A recent review reported a high lifetime prevalence of chronic pain in Europe ranging from 12.7% to 33.7% (35), highlighting its public health importance. Pain has been shown to be associated with both CVD risk factors (7) and incident CVD (36); however, few studies have explored the association between chronic pain and hypertension and their findings are inconsistent (11, 37–39). A very small study demonstrated higher blood pressure among 16 subjects with widespread pain compared with 14 controls (38). A retrospective study of 56,322 primary care patients reported significantly higher risk of elevated blood pressure in patients with severe pain compared with those without pain (odds ratio 1.38, 95% CI: 1.28-1.48) (11). However, this study investigated pain severity rather than chronic pain and did not adjust for potential confounders such as lifestyle, lipid profiles and multimorbidity. Conversely, a bidirectional Mendelian randomization (MR) study showed that genetically predicted essential hypertension was associated with an increased risk of chronic headache (OR 1.007, 95% CI: 1.003–1.011, P=0.002) and limb pain (OR 1.219, 95% CI: 1.033–1.439, P=0.019), but reported no significant association in the reverse direction (39). However, the MR study reported inconsistent findings in the main analysis with all the other sensitivity analyses, with the weighted median analysis, MR Egger, and weighted mode showing no evidence of the bidirectional pain-hypertension relationship. Our findings have extended the evidence of an association between chronic pain and the number of chronic pain and incident hypertension within UK Biobank, showing increased risk of incident hypertension associated with chronic pain, both localized and widespread, and a dose-response relationship, independent of demographic, lifestyle and health confounders.

Musculoskeletal pain is the most common type of chronic pain, affecting 20-50% of adults (40), and is the main cause of disability (41). In spite of these facts, the relationship between musculoskeletal pain and hypertension has rarely been investigated (12, 37, 39). A Mendelian randomization study was non-significant (36) and a cross-sectional study of 17,128 participants reported that chronic low back pain was less frequent among individuals with hypertension compared to those without hypertension (37). However, in common with our findings, a Taiwanese population-based cohort (n=76,304) showed an association between shoulder pain and increased risk of hypertension (12).

Adults with chronic pain have a high prevalence of depression (39.3%, 95% CI, 37.3%-41.1%) (42) and depression is causally associated with developing hypertension (43). Therefore, it is plausible that depression may mediate the association between chronic pain and hypertension. In our study, we found that 11.3% of the association was mediated by depression which is measured by the RDS-4 score. This finding suggests that management of depression in patients with chronic pain may help to reduce their risk of developing hypertension, but additional studies are needed to confirm this finding because the E-value in the sensitivity analysis was less than 1.25, which indicated that the observed mediation effect might be vulnerable to residual confounding. Furthermore, this study also found that CRP partially mediated the association between pain and hypertension, which suggests a potential role of inflammation; however, the mediation effect appears to be small and the E-value in the sensitivity analysis is also quite low, so the mediation effect of inflammation should be interpreted with caution and needs further investigation. Large population studies (44) and systematic reviews and meta-analyses (45) have found raised CRP associated with a diverse range of painful conditions. Pain may lead to the activation of glial and immune cells, releasing pro-inflammatory mediators (20) and inflammation can lead to the development of hypertension through sodium-induced cytokine activation (20). It has previously been suggested that pain medications such as NSAIDs may themselves increase the risk of hypertension (24–26). NSAIDs are the most commonly used for pain management and may affect the blood pressure by antagonizing the anti-hypertensive effect of the drugs or by damaging the renal function. Antidepressant drugs and opioids were also commonly prescribed for chronic pain patients (46), which have been shown to have effect on blood pressure levels through various mechanisms (26, 47). However, previous studies mainly explored the mechanisms behind the change of blood pressure related to medications. Our study conducted the mediation analysis within Cox Hazard regression models by using a large population based cohort study to explore whether the pain-hypertension relation could be explained by the use of these medications. We found no evidence that medications mediated the association between pain and hypertension. However, much more evidence are needed and the findings should be interpreted with caution because pain and medications that were all measured at baseline and the observational study design lead to the unclear causal relationships and the medications adherence and period have not been accounted for.

This study has several strengths including its prospective design, very large sample size and long follow-up, as well as the ability to explore a wide range of pain measures, and adjust for a wide variety of demographic, lifestyle and health confounders. The large effect size (HR) closer to 2.0 for the association between chronic widespread pain and hypertension in the present study would provide stronger evidence for a potential causal relationship, which is important especially under the circumstance that there may be a series of unmeasured confounders and bias. Our study also provided preliminary evidence of possible mechanisms underpinning the association between pain and hypertension.

However, some limitations should be noted. First, the pain characteristics were self-reported using questionnaires and clinical diagnoses of pain such as the ICD-11 definitions of chronic pain or the American College of Rheumatology 1990 (ACR-1990) definition could not be adopted due to their unavailability. Future studies may adopt the objective diagnosis of chronic pain and explore the relation to hypertension. Second, the present study only used the pain characteristics that were measured during one single visit. The lack of repeated measurement of pain for the participants investigated in the present study limits our ability to assess changes in pain over time or confirm the pain classification, particularly for short-term pain. Third, UK Biobank included adults aged 40–69 years of primarily White British origin, so care should be taken in generalizing our findings to other ethnic groups, countries and age groups. A global study showed the high prevalence of pain not only in European populations but other ethnic groups such as African (28%) and Asian (30%) (48). Future studies are warranted to validate the findings in those ethnic groups. Older people had a higher risk of chronic pain than younger people, with prevalence increasing from 14.3% in 18-25 years old to 62% in the over 75 age group (49); it is therefore important to explore whether the association exist in other different age groups, especially the elderly. Forth, in our study, hypertension was defined based on hospital admission and day-case records. However, patients with pain may be more likely to seek medical care, potentially increasing the detection of incident hypertension and introducing surveillance bias. Fifth, no availability of more than two measures of blood pressure limited our ability to understand the relationship between pain and blood pressure trajectories. Therefore, future studies are needed to explore whether chronic pain is related to the longitudinal changes or trajectories in blood pressure. Sixth, the medication as covariates was only assessed at baseline, which may lead to an underestimation of cumulative effects and hindered the evaluation of adherence patterns over time. Finally, although we were able to adjust for a wide range of confounding factors, residual confounding may exist due to the nature of observational studies (50).

## Conclusions

There was a dose-relationship between chronic pain and the development of hypertension, independent of confounders, which was mediated in part by depression and inflammation. Future more studies are warranted to validate the findings between pain and hypertension and explore the potential mediation effects, especially the mediation effect of inflammation due to the small mediation effect. Our findings suggest that consideration might be given to targeting people with chronic pain for the prevention and early detection of hypertension and intervention studies are required to evaluate strategies targeting inflammation and depression.

### Perspectives

Chronic pain was significantly associated with increased risk of the development of hypertension. There was a dose-relationship between chronic pain and the development of hypertension. The association may be mediated in part by depression and inflammation. Future studies are warranted to validate the findings in the other ethnic groups and age groups and to adopt the objective diagnosis of chronic pain and explore the relation to hypertension. The findings implicate that targeting people with chronic pain for the prevention and early detection of hypertension and intervention studies are required to evaluate strategies targeting inflammation and depression.

## Supplementary Material

Supplemental Publication Material

## Figures and Tables

**Table 1:** Baseline characteristics of participants in the UK Biobank

	Overall		Chronic pain			P	Pain type					P for trend
			No		Yes		No pain		Short-term pain	Chronic localized pain	Chronic widespread pain	
N	206963		119523		87440		82869		36654	85118	2322	
Age (years)	53.84 (8.07)		53.65 (8.11)		54.11 (8.01)	<0.001	54.13 (8.09)		52.57 (8.06)	54.09 (8.02)	54.64 (7.67)	<0.001
Men, n (%)	79328 (38.3)		48625 (40.7)		30703 (35.1)	<0.001	33186 (40.0)		15439 (42.1)	30026 (35.3)	677 (29.2)	<0.001
Deprivation						<0.001						<0.001
index, n (%)												
Low	69291 (33.5)		41326 (34.6)		27965 (32.0)		29214 (35.3)		12112 (33.1)	27457 (32.3)	508 (21.9)	
Moderate	69048 (33.4)		40372 (33.8)		28676 (32.8)		28176 (34.0)		12196 (33.3)	28042 (33.0)	634 (27.3)	
High	68365 (33.1)		37685 (31.6)		30680 (35.1)		25386 (30.7)		12299 (33.6)	29502 (34.7)	1178 (50.8)	
Ethnicity: White, n (%)	194284 (96.7)		112561 (96.9)		81723 (96.5)	<0.001	78832 (97.5)		33729 (95.4)	79706 (96.6)	2017 (91.8)	<0.001
Smoking status,						<0.001						
n (%)												
Never	118112 (57.2)		71096 (59.6)		47016 (54.0)		49541 (59.9)		21555 (59.0)	45932 (54.2)	1084 (47.0)	
Previous	64469 (31.2)		36014 (30.2)		28455 (32.7)		25198 (30.5)		10816 (29.6)	27747 (32.7)	708 (30.7)	
Current	23749 (11.5)		12094 (10.1)		11655 (13.4)		7924 (9.6)		4170 (11.4)	11142 (13.1)	513 (22.3)	<0.001
Alcohol (weekly units)	14.04 (15.80)		14.47 (15.53)		13.45 (16.15)	<0.001	14.53 (15.41)		14.33 (15.81)	13.55 (16.13)	9.73 (16.44)	
Physical						<0.001						
activity, n (%)												
Low	30972 (18.1)		17099 (17.1)		13873 (19.6)		11709 (16.8)		5390 (17.7)	13335 (19.3)	538 (32.6)	
Moderate	69753 (40.9)		40923 (40.9)		28830 (40.8)		28409 (40.7)		12514 (41.2)	28261 (41.0)	569 (34.4)	<0.001
High	70012 (41.0)		42101 (42.0)		27911 (39.5)		29619 (42.5)		12482 (41.1)	27366 (39.7)	545 (33.0)	<0.001
Sedentary time (hours), mean (SD)	4.27 (2.56)		4.11 (2.48)	4.48 (2.64)		<0.001	4.03 (2.43)	4.31 (2.58)		4.47 (2.62)	4.97 (3.23)	
Fruit and vegetable intake (portion per day), mean (SD)	4.12 (2.44)	4.14 (2.39)		4.09 (2.51)		<0.001	4.18 (2.39)	4.06 (2.39)		4.09 (2.49)	4.14 (3.10)	
Sleep duration, n (%)						<0.001						
1-6 hours	49041 (23.8)	25081 (21.1)		23960 (27.6)			16776 (20.3)	8305 (22.8)		23098 (27.3)	862 (38.6)	<0.001
7-8 hours	143350 (69.6)	86873 (72.9)		56477 (65.0)			60885 (73.7)	25988 (71.2)		55461 (65.5)	1016 (45.5)	<0.001
≥9 hours	13594 (6.6)	7149 (6.0)		6445 (7.4)			4958 (6.0)	2191 (6.0)		6088 (7.2)	357 (16.0)	<0.001
BMI (kg/m^2^), mean (SD)	26.05 (4.16)	25.69 (3.92)		26.55 (4.42)		<0.001	25.55 (3.85)	26.01 (4.06)		26.52 (4.39)	27.84 (5.20)	
Waist (cm), mean (SD)	86.07 (12.20)	85.38 (11.89)		87.02 (12.55)		<0.001	84.97 (11.80)	86.28 (12.05)		86.93 (12.51)	90.00 (13.61)	
HDL (mmol/L), mean (SD)	1.49 (0.38)	1.50 (0.38)		1.47 (0.38)		<0.001	1.52 (0.39)	1.46 (0.37)		1.47 (0.38)	1.41 (0.37)	
TG (mmol/L), mean (SD)	1.57 (0.93)	1.53 (0.90)		1.62 (0.96)		<0.001	1.51 (0.89)	1.57 (0.93)		1.62 (0.96)	1.79 (1.06)	<0.001
LDL (mmol/L), mean (SD)	3.55 (0.83)	3.54 (0.82)		3.57 (0.84)		<0.001	3.54 (0.82)	3.53 (0.82)		3.57 (0.83)	3.54 (0.88)	<0.001
Total Cholesterol (mmol/L),	5.69 (1.08)	5.68 (1.07)		5.71 (1.09)		<0.001	5.70 (1.07)	5.64 (1.06)		5.71 (1.09)	5.64 (1.16)	<0.001
mean (SD) CRP_0 (mg/L), mean (SD)	2.17 (4.01)	1.99 (3.78)		2.42 (4.29)		<0.001	1.89 (3.55)	2.22 (4.24)		2.37 (4.18)	4.27 (7.12)	<0.001
HbAlc 0 (mmol/mol), mean (SD)	34.94 (5.41)		34.81 (5.14)		35.12 (5.76)	<0.001	34.81 (5.10)		34.81 (5.22)	35.08 (5.71)	36.40 (7.24)	<0.001
SBP (mmHg), mean (SD)	123.80 (9.88)		123.96 (9.86)		123.58 (9.91)	<0.001	124.07 (9.84)		123.70 (9.89)	123.60 (9.90)	123.00 (10.25)	<0.001
DBP (mmHg), mean (SD) Number of long-term conditions, n (%)	76.12 (7.02)		76.05 (7.03)		76.22 (7.01)	<0.001 <0.001	75.94 (7.04)		76.31 (7.00)	76.21 (7.00)	76.40 (7.21)	<0.001 <0.001
	
0	98191 (47.4)		66914 (56.0)		31277 (35.8)		47359 (57.1)		19555 (53.4)	30959 (36.4)	318 (13.7)	
1	67581 (32.7)		36757 (30.8)		30824 (35.3)		25147 (30.3)		11610 (31.7)	30107 (35.4)	717 (30.9)	
≥2	41191 (19.9)		15852 (13.3)		25339 (29.0)		10363 (12.5)		5489 (15.0)	24052 (28.3)	1287 (55.4)	
Depression score (RDS-4)	5.51 (2.16)		5.14 (1.83)		6.00 (2.46)	<0.001	5.00 (1.70)		5.47 (2.06)	5.95 (2.41)	7.81 (3.38)	<0.001
Insulin therapy, n (%)	404 (0.2)		226 (0.2)		178 (0.2)	0.492	156 (0.2)		70 (0.2)	170 (0.2)	8 (0.3)	<0.001
Cholesterol-lowering medications, n (%)	16653 (8.0)		8530 (7.1)		8123 (9.3)	<0.001	5885 (7.1)		2645 (7.2)	7733 (9.1)	390 (16.8)	<0.001
Anti-depressive medications, n (%)	8051 (3.9)		3119 (2.6)		4932 (5.6)	<0.001	1978 (2.4)		1141 (3.1)	4574 (5.4)	358 (15.4)	<0.001
Aspirin, n (%)	16202 (7.8)		8123 (6.8)		8079 (9.2)	<0.001	5421 (6.5)		2702 (7.4)	7785 (9.1)	294 (12.7)	<0.001
Opioid, n (%)	2820 (2.1)		456 (0.7)		2364 (3.6)	<0.001	243 (0.5)		213 (0.9)	2203 (3.4)	161 (7.6)	<0.001
NSAIDs, n (%)	34540 (16.7)		14367 (12.0)		20173 (23.1)	<0.001	8536 (10.3)		5831 (15.9)	19330 (22.7)	843 (36.3)	<0.001

BMI, body mass index; DBP, diastolic blood pressure; HDL, high-density lipoprotein; HbA1c, glycated haemoglobin; LDL, low-density lipoprotein; NSAID, nonsteroidal anti-inflammatory drugs; RDS, recent depressive symptoms; SBP, systolic blood pressure; SD, standard deviation; TG, triglyceride; WC, waist circumference.

**Table 2:** Association of pain type and incident hypertension

	Model 1		Model 2	Model 3		Model 4	
Pain type	HR	95% CI	HR	95% CI	HR	95% CI	HR	95% CI
No pain	ref		ref		ref		ref	
Short-term pain	1.19	1.14, 1.24	1.11	1.05, 1.16	1.09	1.03, 1.16	1.10	1.03, 1.17
Chronic localized pain	1.62	1.57, 1.67	1.38	1.33, 1.43	1.22	1.17, 1.28	1.20	1.14, 1.26
Chronic widespread pain	3.10	2.82, 3.40	2.25	2.00, 2.53	1.84	1.62, 2.10	1.75	1.52, 2.00
Chronic pain (no/yes)	1.57	1.53, 1.62	1.36	1.31, 1.40	1.20	1.16, 1.25	1.18	1.13, 1.23

Abbreviations: Ref, reference group; BMI, body mass index; CI, confidence interval; HDL, high-density lipoprotein cholesterol; HbA1c, hemoglobin A1C; HR, hazard ratio; SBP, systolic blood pressure; WC, waist circumference; NSAID, nonsteroidal anti-inflammatory drugs.

Model 1: adjusted for age, sex, Townsend deprivation index, and ethnicity;

Model 2: additionally adjusted for smoking status, weekly units of alcohol use, physical activity, total sedentary time, sleep duration, fruit and vegetable intake, BMI, and WC;

Model 3: additionally adjusted for HDL, total cholesterol, SBP, HbA1c, and number of long-term conditions, use of cholesterol-lowering medications, and insulin therapy.

Model 4: additionally adjusted for use of antidepressant medications, aspirin, opioid, and NSAIDs.

**Table 3:** Association of the number of chronic pain and chronic musculoskeletal pain and incident hypertension

	Model 1		Model 2		Model 3		Model 4	
	HR	95% CI	HR	95% CI	HR	95% CI	HR	95% CI
**Chronic pain** **number **0	ref		ref		ref		ref	
1	1.30	1.26, 1.35	1.19	1.14, 1.24	1.1	1.05, 1.15	1.09	1.03, 1.15
2–3	1.74	1.68, 1.80	1.49	1.43, 1.56	1.29	1.22, 1.35	1.24	1.17, 1.31
≥ 4	2.53	2.36, 2.70	1.80	1.66, 1.97	1.39	1.26, 1.53	1.31	1.19, 1.45
Chronic widespread pain (no/yes)	3.00	2.74, 3.29	2.23	1.98, 2.51	1.81	1.59, 2.07	1.73	1.50, 1.99
Chronic pain number (per site)	1.24	1.22, 1.25	1.16	1.14, 1.17	1.09	1.07, 1.10	1.07	1.05, 1.09
P for trend	<0.001		<0.001		<0.001		<0.001	
**Chronic musculoskeleta l pain**								
0	ref		ref		ref		ref	
1	1.33	1.29, 1.38	1.20	1.15, 1.25	1.12	1.07, 1.17	1.11	1.06, 1.17
2-3	1.80	1.74, 1.87	1.51	1.44, 1.58	1.28	1.21, 1.35	1.25	1.19, 1.33
4	2.39	2.17, 2.64	1.52	1.33, 1.73	1.19	1.03, 1.38	1.12	0.97, 1.31
Chronic musculoskeletal pain (no/yes)	1.48	1.44, 1.53	1.29	1.24, 1.33	1.15	1.11, 1.20	1.14	1.09, 1.19
Chronic musculoskeletal pain number (per site)	1.27	1.25, 1.28	1.16	1.14, 1.18	1.09	1.06, 1.11	1.07	1.05, 1.10
P for trend	<0.001		<0.001		<0.001		<0.001	

Abbreviations: Ref, reference group; BMI, body mass index; CI, confidence interval; HDL, high-density lipoprotein cholesterol; HbA1c, hemoglobin A1C; HR, hazard ratio; SBP, systolic blood pressure; WC, waist circumference; NSAID, nonsteroidal anti-inflammatory drugs.

Model 1: adjusted for age, sex, Townsend deprivation index, and ethnicity;

Model 2: additionally adjusted for smoking status, weekly units of alcohol use, physical activity, total sedentary time, sleep duration, fruit and vegetable intake, BMI, and WC;

Model 3: additionally adjusted for HDL, total cholesterol, SBP, HbA1c, and number of long-term conditions, use of cholesterol-lowering medications, and insulin therapy.

Model 4: additionally adjusted for use of antidepressant medications, aspirin, opioid, and NSAIDs.

**Table 4:** Association of different painful sites and the risk of incident hypertension

Painful sites		Model 1		Model 2		Model 3		Model 4	
		HR	95% CI	HR	95% CI	HR	95% CI	HR	95% CI
No pain		ref		ref		ref		ref	
Headaches	Short-term	1.43	1.37, 1.51	1.26	1.18, 1.33	1.17	1.09, 1.25	1.15	1.07, 1.23
	Chronic	1.73	1.64, 1.82	1.52	1.43, 1.61	1.30	1.22, 1.39	1.22	1.13, 1.31
Facial	Short-term	1.37	1.10, 1.70	1.15	0.88, 1.51	1.08	0.79, 1.46	1.15	0.84, 1.59
	Chronic	2.07	1.69, 2.54	1.60	1.23, 2.08	1.20	0.90, 1.62	1.21	0.90, 1.64
Neck/shoulder	Short-term	1.36	1.27, 1.46	1.19	1.10, 1.29	1.14	1.04, 1.25	1.15	1.04, 1.28
	Chronic	1.62	1.54, 1.70	1.40	1.32, 1.48	1.21	1.13, 1.29	1.19	1.11, 1.28
Back	Short-term	1.30	1.21, 1.39	1.21	1.12, 1.32	1.21	1.11, 1.32	1.24	1.12, 1.37
	Chronic	1.49	1.41, 1.58	1.25	1.17, 1.34	1.14	1.06, 1.22	1.16	1.07, 1.25
Abdominal	Short-term	1.43	1.25, 1.64	1.29	1.10, 1.51	1.16	0.97, 1.38	1.13	0.92, 1.38
	Chronic	1.68	1.48, 1.91	1.64	1.42, 1.90	1.43	1.22, 1.68	1.43	1.20, 1.71
Hip	Short-term	1.33	1.12, 1.58	1.14	0.92, 1.41	1.09	0.86, 1.36	1.10	0.86, 1.42
	Chronic	1.52	1.38, 1.68	1.31	1.17, 1.47	1.19	1.05, 1.35	1.17	1.01, 1.34
Knee	Short-term	1.13	1.00, 1.29	1.05	0.90, 1.21	1.11	0.94, 1.30	1.12	0.93, 1.35
	Chronic	1.34	1.25, 1.44	1.14	1.05, 1.24	1.07	0.98, 1.18	1.08	0.97, 1.20
Widespread	Short-term	1.31	0.99, 1.72	1.02	0.72, 1.43	1.03	0.72, 1.48	1.10	0.76, 1.60
pain	Chronic	3.10	2.83, 3.41	2.25	2.00, 2.54	1.84	1.62, 2.10	1.74	1.52, 2.00

Abbreviations: Ref, reference group; BMI, body mass index; CI, confidence interval; HDL, high-density lipoprotein cholesterol; hemoglobin A1C; HR, hazard ratio; SBP, systolic blood pressure; WC, waist circumference; NSAID, nonsteroidal anti-inflammatory drugs.

Model 1: adjusted for age, sex, Townsend deprivation index, and ethnicity;

Model 2: additionally adjusted for smoking status, weekly units of alcohol use, physical activity, total sedentary time, sleep duration, fruit and vegetable intake, BMI, and WC;

Model 3: additionally adjusted for HDL, total cholesterol, SBP, HbA1c, and number of long-term conditions, use of cholesterol-lowering medications, and insulin therapy.

Model 4: additionally adjusted for use of antidepressant medications, aspirin, opioid, and NSAIDs.

**Table 5:** Exploration of potential mediators in the association of pain and incident hypertension

Outcome	Total effect	Natural Direct effect	Natural Indirect effect	Proportion mediated, % (95% CI)
	HR (95% CI)	HR (95% CI)	HR (95% CI)	
**Single mediator**				
Depression	1.175 (1.128, 1.229)	1.155 (1.108, 1.207)	1.017 (1.010, 1.023)	11.30 (6.38, 17.60)
CRP	1.177 (1.132, 1.235)	1.176 (1.132, 1.234)	1.0006 (1.00008, 1.001)	0.40 (0.06, 0.90)
NSAIDs	1.179 (1.128, 1.230)	1.179 (1.128, 1.230)	0.999 (0.996 1.004)	-0.53 (-0.31, 2.50)
Antidepress ants	1.178 (1.128, 1.230)	1.178 (1.128, 1.230)	0.999 (0.999 1.001)	-0.01 (-0.47, 0.70)
Aspirin	1.185 (1.138, 1.230)	1.185 (1.138, 1.230)	0.998 (0.996 1.009)	-1.40 (-2.60, 5.60)
Opioid	1.178 (1.130, 1.228)	1.178 (1.130, 1.228)	1.000 (0.996 1.004)	0.05 (-2.70, 2.90)
**Multiple mediators (CRP+depr ession)**				
Total mediation	1.175 (1.132, 1.227)	1.157 (1.113, 1.209)	1.018 (1.012, 1.024)	11.70 (7.65, 17.40)

CI, confidence interval; CRP, C-reactive protein; HR, hazard ratio; NSAID, nonsteroidal anti-inflammatory drugs.

Model adjusted for age, sex, Townsend deprivation index, ethnicity, smoking status, weekly units of alcohol use, physical activity, total sedentary time, sleep duration, fruit and vegetable intake, BMI, WC, HDL, total cholesterol, SBP, HbA1c, and number of long-term conditions, use of cholesterol-lowering medications, and insulin therapy.

## Data Availability

Data analysed in this study was from the UK Biobank and is available upon application to UK Biobank https://www.ukbiobank.ac.uk.

## Pathophysiological Novelty and Relevance What is new?

Evidence is lacking on the associations between pain type, duration and location and hypertension and no study has explored the dose-relationship between the number of chronic pain sites and incident hypertension. To our knowledge, the present study is the first to uncover the relationship between different pain characteristics (pain type, location and spread) and incident hypertension, and whether they were mediated by inflammation, depression or medication in a large-scale population cohort. Chronic pain was associated with incident hypertension, independent of demographic, lifestyle, and health confounders. Consistent dose-response relationships were found in terms of the number of body sites affected by chronic pain and chronic musculoskeletal pain, and across the spectrum from no pain, short-term pain, and chronic localized pain to chronic widespread pain.

## What is Relevant?

Understanding the relationship between chronic pain and hypertension may identify new mechanisms and targets to prevent and treat hypertension.

## Clinical/Pathophysiological Implications?

These findings suggest chronic pain is a risk factor of hypertension and depression and inflammation may account for a large part of the association. Consideration should be given to targeting people with chronic pain for the prevention and early detection of hypertension and intervention studies are required to evaluate strategies targeting inflammation and depression.
