# Supplementary material for: Chronic pain and hypertension and mediation role of inflammation and depression
Source: Hypertension. Author manuscript; Available in PMC 2026 Jan 1. (PMC7618362; doi:10.1161/HYPERTENSIONAHA.125.25544)
Supplement: Supplemental Publication Material [file EMS209042-supplement-Supplemental_Publication_Material.docx]

**Chronic pain and hypertension and mediation role of inflammation and depression**

**Running title: Chronic pain and hypertension**

Pei Qin^1^, Frederick K. Ho^1^, Carlos A. Celis-Morales^2,3,4^, Jill P. Pell^1*^

^1^ School of Health and Wellbeing, University of Glasgow, Glasgow UK.

^3^ School of Cardiovascular and Metabolic Health, University of Glasgow, Glasgow UK.

^4^ Human Performance Lab, Education, Physical Activity and Health Research Unit, University Católica del Maule, Talca, Chile.

^5^ Centro de Investigación en Medicina de Altura (CEIMA), Universidad Arturo Prat, Iquique, Chile.

***Corresponding Author：**

Professor Jill Pell CBE

School of Health and Wellbeing, Clarice Pears Building, 90 Byres Road, University of Glasgow, G12 8TB, United Kingdom

Email: [Jill.Pell@glasgow.ac.uk](mailto:Jill.Pell@glasgow.ac.uk)

**Table S1 Sensitivity analysis on the mediation effect considering unmeasured confounding**

| **Outcome** | **Total effect** |  | **Natural Direct effect** |  | **Natural Indirect effect** |
| --- | --- | --- | --- | --- | --- |
|  | **E-value point estimate (CI)** |  | **E-value point estimate (CI)** |  | **E-value point**  **estimate (CI)** |
| **Single mediator** |  |  |  |  |  |
| Depression | 1.628 (1.508) |  | 1.578 (1.454) |  | 1.148 (1.110) |
| CRP | 1.633(1.519) |  | 1.631 (1.519) |  | 1.025 (1.009) |
| NSAIDs | 1.638 (1.508) |  | 1.638 (1.508) |  | 1.033 (1.000) |
| Antidepressants | 1.638 (1.508) |  | 1.638 (1.508) |  | 1.033 (1.000) |
| Aspirin | 1.653 (1.534) |  | 1.653 (1.534) |  | 1.047 (1.000) |
| Opioid | 1.636 (1.513) |  | 1.636 (1.513) |  | 1.000 (1.000) |
| **Multiple mediators (CRP+depression)** |  |  |  |  |  |
| Total mediation | 1.628 (1.519) |  | 1.583 (1.468) |  | 1.153 (1.122) |

CI, confidence interval; CRP, C-reactive protein; HR, hazard ratio; NSAID, nonsteroidal anti-inflammatory drugs.

Model adjusted for age, sex, Townsend deprivation index, ethnicity, smoking status, weekly units of alcohol use, physical activity, total sedentary time, sleep duration, fruit and vegetable intake, BMI, WC, HDL, total cholesterol, SBP, HbA1c, and number of long-term conditions, use of cholesterol-lowering medications, and insulin therapy.
